# Investigation of Heterogeneity Sources for Occupational Task Recognition via Transfer Learning

**DOI:** 10.3390/s21196677

**Published:** 2021-10-08

**Authors:** Sahand Hajifar, Saeb Ragani Lamooki, Lora A. Cavuoto, Fadel M. Megahed, Hongyue Sun

**Affiliations:** 1 Department of Industrial & Systems Engineering, University at Buffalo, Buffalo, NY 14260, USA; sahandha@buffalo.edu (S.H.); loracavu@buffalo.edu (L.A.C.); 2 Department of Mechanical and Aerospace Engineering, University at Buffalo, Buffalo, NY 14260, USA; saebraga@buffalo.edu; 3 Farmer School of Business, Miami University, Oxford, OH 45056, USA; fmegahed@miamioh.edu

**Keywords:** occupational human activity recognition, domain adaptation, transfer learning, wearable sensors

## Abstract

Human activity recognition has been extensively used for the classification of occupational tasks. Existing activity recognition approaches perform well when training and testing data follow an identical distribution. However, in the real world, this condition may be violated due to existing heterogeneities among training and testing data, which results in degradation of classification performance. This study aims to investigate the impact of four heterogeneity sources, cross-sensor, cross-subject, joint cross-sensor and cross-subject, and cross-scenario heterogeneities, on classification performance. To that end, two experiments called separate task scenario and mixed task scenario were conducted to simulate tasks of electrical line workers under various heterogeneity sources. Furthermore, a support vector machine classifier equipped with domain adaptation was used to classify the tasks and benchmarked against a standard support vector machine baseline. Our results demonstrated that the support vector machine equipped with domain adaptation outperformed the baseline for cross-sensor, joint cross-subject and cross-sensor, and cross-subject cases, while the performance of support vector machine equipped with domain adaptation was not better than that of the baseline for cross-scenario case. Therefore, it is of great importance to investigate the impact of heterogeneity sources on classification performance and if needed, leverage domain adaptation methods to improve the performance.

## 1. Introduction

The Operator 4.0 implementation has continued to grow at unprecedented rates. It represents “a new design and engineering philosophy for adaptive production systems where the focus is on treating automation as a further enhancement of the human’s physical, sensorial, and cognitive capabilities” [1]. Legacy systems, cognitive healthcare, maintenance and prediction, and machine-to-people (M2P) interaction based on operator position are among the important applications of Operator 4.0 [2,3]. Among these applications, cognitive healthcare involves healthy operator and smarter operator typologies [2]. Maintenance and prediction as well as M2P interaction based on operator position also require smarter operator typology [2]. A healthy operator uses a wearable tracker by which his/her health-related metrics are monitored and his/her sudden movements (e.g., fall of the operator) are detected [2,4,5,6]. Smarter operator, on the other hand, provides intelligent personal assistant to the operator [2]. Thus, it is evident that human activity recognition (HAR) can directly/indirectly facilitate realization of Operator 4.0 in a workplace. In particular, in an Operator 4.0-compliant workplace, HAR is required to prevent potential threats that adversely affect the safety and production quality through monitoring health-related indicators and informing administrators when required [7,8].

HAR has been extensively used in occupational environments. For instance, Nath et al. [9] employed body-mounted smartphones to collect time-stamped motion data from construction workers and extract duration and frequency of labor-intensive activities, which were then categorized into ergonomic risk levels. Similarly, Zhang et al. [10] used HAR to manage and monitor floor-reinforcing steel workers, and eventually, control cost, quality, progress, and safety through process management, i.e., positioning workers, material and equipment. In another study, a wrist-worn wearable sensor was attached to the dominant wrist of workers to detect assembly tasks performed by them [11]. This information can then be used to provide proactive instructions or verify that all the required work steps are performed [11].

However, in real-world applications, HAR performance can be adversely affected by heterogeneity sources (e.g., variety of workers, different types of sensors) within a certain occupation. This occurs when the unseen testing data (e.g., data from a subject or a type of sensor) significantly differs from those used to train HAR models (e.g., data from another subject or another type of sensor). Here, the focus is to investigate the impact of potential heterogeneity sources on the HAR of a common set of activities, so that the HAR of these activities can be generalized. The potential heterogeneities in occupation settings include sensing heterogeneities, including sensor biases, sampling instabilities and different sampling rates [12], environmental heterogeneities (for instance, heterogeneity between controlled lab environment and real-world [13]), subject heterogeneities (for instance, fitness level, gender and body structure heterogeneities [14]), and others that may arise. In particular, cross-sensor heterogeneities can be resulted by modifications in the configuration and type of sensors due to different preferences and requirements by different subjects and workplaces, respectively [15]. Environmental heterogeneities can be caused by variability of the individuals’ physical activities when performed in a controlled lab environment versus in-the-wild [16]. Finally, subject heterogeneities exist, as in practice, HAR methods are usually applied to unseen subjects with different fitness level, gender and body structure [14]. Most of the existing literature either neglects to consider these heterogeneities or investigates them individually, with a great emphasis placed on cross-subject heterogeneity. Once the type of heterogeneity is identified and acknowledged to be problematic for HAR, the next step is to take appropriate actions toward alleviating the problem.

Depending on the heterogeneity, existing studies have used various approaches to deal with the challenges discussed above, including active learning, deep learning, field experiments and domain adaptation. Here, we will describe how active learning, deep learning and field experiments can mitigate the heterogeneity issue, why their usability is limited in an industrial environment, and elaborate on why domain adaptation is more favorable.

Environmental heterogeneity and the wide range of task performance approaches by different subjects negatively impacts the large-scale deployment of HAR systems [17]. Active learning has been proposed to partially solve this problem by querying the tasks being performed by new subjects/in new environments and generating customized models [17]. For example, heterogeneities in speed of walking, gestures and sleep habits may require personalized models for individual users [18]. To this end, Hossain et al. [18] capitalized on active learning to enhance the recognition of activities of daily living (ADLs), where the users were actively queried for labelling the activities. The usability of active learning would be limited for industrial environments due to the interfering nature of querying. Firstly, HAR systems must continuously adapt to the new needs of fast-changing industrial environments; therefore, repetitive queries can place a large burden on users [19]. Secondly, excessive queries can distract workers and workplace distraction has an adverse impact on hazard recognition, safety risk perception, and safety performance [20].

Due to outstanding success achieved by deep learning in image classification, researchers have been motivated to transform time series data into an image structure, using methods such as Recurrence Plot (RP), Markov Transition Field (MTF), and Gramian Angular Fields (GAF), and approach the HAR problem from a computer vision viewpoint [21]. For instance, Abdel-Basset et al. [22] leveraged deep learning approaches to perform activity recognition using heterogeneous inertial sensors. Their proposed approach was able to achieve accuracies of 98% and 99% when applied to two public heterogeneous HAR (HHAR) datasets (i.e., HHAR UCI [23] and mHEALTH [24]). HHAR UCI and mHEALTH datasets are comprised of 14,299,880 and 343,195 labeled ADL instances, respectively. Deep learning algorithms require such huge datasets to learn latent patterns [25], which are usually unattainable in industrial applications due to data scarcity.

Sometimes studies are more concerned about the situations that subjects experience in their natural environment and thus a field experiment is preferred [26]. A field experiment is performed in the wild and the activities performed in this type of experiment are a good representation of real-world activities [9]. Moreover, the participants are allowed to perform the activities at their own comfortable pace [9]. HAR based on field experiments has been particularly promising in the areas of healthcare and nursing care facilities [27,28]. However, one must be cautious when performing field experiments because the lack of control and failure of accurately characterizing the field environment may adversely affect the generalizability of the study [29]. Moreover, the results of field and lab-based testing have been shown to be comparable under favorable operational conditions [30,31].

Alternatively, unsupervised domain adaptation (UDA) has gained increasing popularity in HHAR [32,33]. UDA can transfer the information learnt from an annotated source data (training data) to an unannotated target data (test data) such that time-consuming data recollections can be eliminated [34]. Here, the source data and target data correspond to different subjects/sensors/environments, dependent on the heterogeneity sources. We want to build an HAR approach that can generalize in the heterogeneous settings, e.g., from one sensor to another or from a lab environment to the wild, based on UDA. Owing to its unsupervised nature, UDA does not require any querying for labelling the activities from target data and consequently, does not place any additional burden on subjects.

Existing literature has shown that UDA is effective for reusing existing knowledge to classify a set of ADLs and sport activities performed by heterogeneous subjects, sensors and environmental situations [35,36]. In a sports dataset that included walking, jogging, cycling, going upstairs and going downstairs, UDA resulted in at least 4.8% and 9.3% improvements in the classification accuracies of cross-subject and cross-sensor scenarios, respectively [36]. In an ADL context comprised of sitting, standing, walking and running activities, a multi-source UDA method achieved 2.0% improvement in the cross-subject classification accuracy [37]. In these and related studies, accuracy improvements usually varied based on the type of the heterogeneity under study.

To promote the transferability of activity recognition models in classifying a common set of activities under heterogeneity sources, there is a lack of a comprehensive study of potential heterogeneities in an occupational context and their impact on HAR performance. This study aims to analyze four potential heterogeneities in the occupational context of electrical line workers (ELWs), including cross-sensor, cross-subject, joint cross-sensor and cross-subject, and cross-scenario heterogeneities. We focus on ELWs, as they are often lone workers lacking direct supervision, where an automatic monitoring is of great importance. Here, we elaborate on these heterogeneities to address four research questions:

1. Cross-sensor:
  1.1. Can the information learned from an existing wearable sensor be directly used to perform activity recognition for the same set of activities collected by a new wearable sensor?
  1.2. If the answer is no, can we transfer the information learned from the existing wearable sensor to the new one and use the new wearable sensor to classify the activities that were detectable by the old sensor?
  This transferability eliminates the need for collecting a large amount of data on new sensors and retraining machine learning algorithms when any changes or updates occur in configuration of the system, such as replacing a sensor.
2. Cross-subject:
  2.1. Can the models trained using a set of activities performed by a limited number of subjects be used to recognize the same set of activities for a new unseen subject?
  2.2. Can (and by how much can) the performance of activity recognition be improved by transferring the information learned from existing subjects (or a subset of existing subjects) to a new subject?
  Answering these questions will inform us of whether (and how) we can circumvent collecting labelled data from every single new subject, which is burdensome and in some cases, infeasible.
3. Cross-sensor and cross-subject: How does a combination of cross-sensor and cross-subject heterogeneities impact the model performance for classifying a common set of activities?
  This question focuses on the more complex scenario that more than one potential heterogeneity sources exists, and whether the information can be transferred.
4. Cross-scenario: Can (and how can) the information learned for classifying a common set of activities from a controlled lab experiment be applied/transferred to a real-world environment?
  Most activity recognition experiments are performed in a controlled lab environment; however, the environmental situation of a real-world scenario might be different from that of a controlled lab experiment. For instance, in lab research, a subject might be asked to consecutively ascend and descend a ladder with a certain number of repetitions in order to facilitate the data collection and annotation (labelling) process. However, in a real-world environment, sporadic occurrences of ascending-descending repetitions are more common. Answering this question focuses on how such heterogeneities impact the performance of activity recognition.

**Figure 1:**
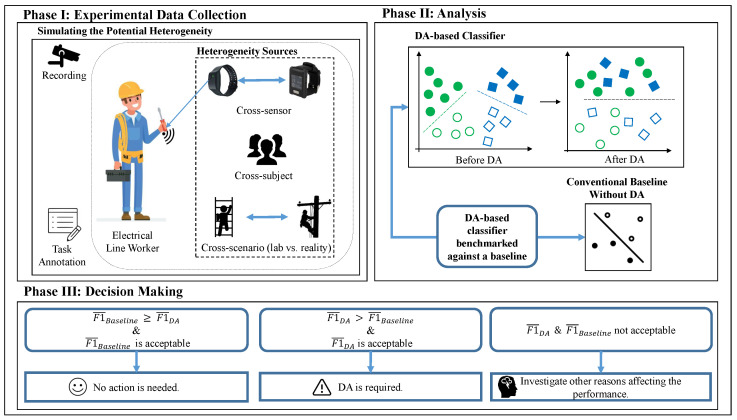
An overview of the proposed framework (${\bar{F1}}_{Baseline}$ and ${\bar{F1}}_{DA}$ represent overall F1 score obtained from a SVM classifier and a SVM classifier equipped with DA, respectively.)

To address these questions, we will capitalize on transfer learning and compare it to the case of applying standard machine learning models. Figure 1 presents an overview of our framework in three phases. In Phase I, the occupational environment of electrical line workers is simulated, along with the mentioned heterogeneities. In Phase II, the classification is performed using a DA-based classifier and a conventional classifier and the two methods are benchmarked against each other. Finally, in Phase III, decision makers rely on the comparative analysis results obtained from Phase II to take actions.

**Figure 2:**
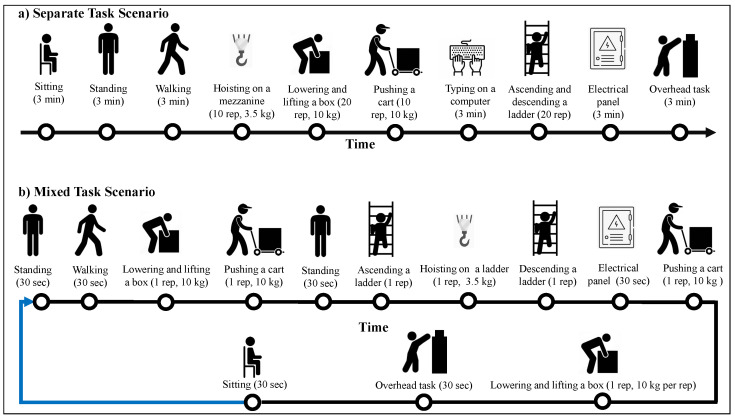
The timeline of experimental tasks.

In particular, in Phase I, the simulated experiment involves fine motor skill activities, such as electrical panel work, overhead tasks and typing on a computer, and gross motor activities, such as hoisting a weighted bucket from a mezzanine/ladder, lowering and lifting a box, pushing a cart and ascending and descending a ladder, which are commonly performed by electrical line workers, along with activities of daily living such as sitting, standing and walking. The sources of heterogeneities include cross-sensor heterogeneity, such as bias between different sensors and inconsistent sampling rates, cross-subject heterogeneity, such as variations in lifestyle and health status of the subjects, joint cross-sensor and cross-subject heterogeneity, and cross-scenario heterogeneity, such as variations in dispersion and duration of the tasks. Triaxial acceleration data were collected from two wristbands, Empatica E4 and Maxim, worn by 18 subjects. For the cross-scenario (environmental heterogeneity), we simulated two lab experiments called separate task scenario and mixed task scenario (see more details in Section 3.1, Figure 2). The design of the separate task scenario is similar to that of most activity recognition experiments, where each task is performed separately and then followed by the next task. On the other hand, the mixed task scenario was designed to mimic a real-world environment, where the tasks were performed sporadically and spread over the time of the experiment as single repetitions (for repetition-based tasks) and for shorter durations (for time-based tasks). The separate task scenario experiment will be used to answer questions 1–3, while both experiments will be used to answer question 4.

In Phase II, we use a UDA method, geodesic flow kernel (GFK), to transfer the information from the source domain (e.g., one subject/one sensor type/separate experiment) to the target domain (e.g., another subject/another sensor type/mixed experiment) for all four questions. The time-frequency features of the wearable sensors, calculated based on discrete wavelet transform were input to GFK. When multiple subjects are involved (as in questions 2 and 3), we use the Rank of Domain (ROD) metric to determine which existing subjects would give us the best performance on the new unseen subject without needing to run the GFK algorithm and building classifiers [38].

In Phase III, given the identified significant heterogeneity sources (second column in Phase III of Figure 1), one would use DA to alleviate the heterogeneity issue and improve performance. If the heterogeneity is not significant (first column of Phase III in Figure 1), a conventional classifier can be used to recognize the activities. Finally, if a factor other than heterogeneity negatively impacts the activity recognition performance (third column of Phase III in Figure 1), further root cause analysis is needed to identify the harmful factor.

## 2. Background and Literature Review

### 2.1. Domain Adaptation

DA allows solving a machine learning problem in the target (test) domain by using the data in the source (training) domain, when these domains have heterogeneous but related distributions [39]. Therefore, DA obviates the need for costly data labelling and retraining machine learning models when dealing with a new unseen domain [39]. Within the field of DA, there are two main types of approaches: semi-supervised and unsupervised. The major difference between the two types of DA is that semi-supervised DA requires a limited number of the target data to be labelled [40,41,42,43]. Unsupervised DA, on the other hand, does not need any observation from the target data to be labelled [44,45,46,47]. We limit our focus to unsupervised DA because in our problem setting no activity labels are available for a new unseen subject. Three major approaches have been adopted to solve unsupervised DA. The first approach solves unsupervised DA by aligning the feature distributions in the source domain with the target domain using a certain metric [48,49]. The second approach solves the same problem through selecting the observations from the source domain or reassigning weights to them [50,51], while the third approach aims to learn a specific feature space transformation that can map the distribution of the source domain to that of the target domain [52,53]. Due to the heterogeneities inherent in HAR, DA has gained increasing attention in this field. We review HAR based on DA studies in Section 2.2.

### 2.2. HAR Based on Domain Adaptation

As discussed in the introduction, HAR based on DA studies can be categorized according to the type of heterogeneity under study. To be more precise, here, we review the most related ones to our work, including cross-sensor and cross-subject heterogeneities.

For cross-sensor DA, deep DA has been widely used, which can be categorized into two categories, i.e., discrepancy measurement and adversarial learning [36] (please note that an adversarial learning approach may also employ discrepancy measures). For the former, Akbari and Jafari [15] developed an unsupervised DA algorithm based on deep learning that aims to minimize the distributional discrepancy between two sets of features extracted from two wearable sensors. Their algorithm only used the source data (smartwatch/smartphone) and a limited number of unlabelled samples from the target data (smartphone/smartwatch) and was able to outperform the state-of-the-art DA algorithms when applied to an ADL dataset (HHAR dataset). While for the latter, Zhou et al. [36] proposed a novel adversarial deep domain adaptation framework that first determines and selects the most relevant source datasets and then obtains the sensor invariant features. To achieve a cross-sensor problem setting, they considered 4 different devices, including Huawei Watch, Huami Watch, Mi Band and Huawei Nexus, and showed that their algorithm was able to improve the classification accuracy of gesture and sports activities.

HAR often suffers poor transferability from one subject to another. Therefore, cross-subject DA has been the concern of several studies focusing on two categories of discrepancy measurement and adversarial learning. For discrepancy measurement, Hosseini et al. [54] and Zhao et al. [55] studied deep features and shallow features, respectively. In particular, Hosseini et al. [54] employed a deep DA approach equipped with a Mean Maximum Discrepancy (MMD) discrepancy loss to transfer ADL recognition models learned based on adult subjects to children. They demonstrated that the F1 score of their proposed approach was 9.0% lower than the F1 score of a supervised baseline, which was more promising than an unsupervised baseline without DA with a 25.2% reduction in F1 score. Similarly, Zhao et al. [55] used the MMD discrepancy loss, but at a finer granularity. In particular, they tackled this problem by developing a 3-step algorithm called local domain adaptation (LDA). In their algorithm, firstly, the activities were grouped into multiple high-level clusters. Secondly, each cluster from the source domain and its corresponding cluster from the target domain were aligned in a low-dimensional subspace. Finally, the labels of the target domain were predicted using the features in a low-dimensional subspace. They verified the effectiveness of LDA by applying it to two widely-used ADL and sports datasets. For deep adversarial DA, Zhou et al. [36] and Chakma et al. [37] studied multi-source setting using two different approaches. Zhou et al. [36] employed an adversarial deep DA approach to transfer the models between different groups of subjects, where the subjects were grouped based on sex, age and body mass index (BMI). They developed a domain relevance calculator that can select the best source domain for adaptation. Their proposed approach resulted in at least 4.8% improvement in the classification accuracy. Chakma et al. [37] proposed a deep Multi-source Adversarial Domain Adaptation (MSADA) framework that extracts a feature representation in which all of the domains (multiple sources and one target) become as relevant as possible. Unlike Zhou et al. [36], their approach is capable of learning a domain invariant feature across multiple domains, rather than selecting the best source. They showed that MSADA approach achieves 2% improvement in accuracy when applied to the cross-person problem of OPPORTUNITY dataset [56] with four ADLs. Finally, Ding et al. [57] carried out a study on unsupervised DA between different subjects by comparing several discrepancy measurement and adversarial learning state-of-the-art DA algorithms. They found that the MMD method developed by Pan et al. [58] is the most appropriate method for HAR.

We have summarized the areas, heterogeneity sources, source data, target data and accuracy improvement results from a number of studies that applied DA to HAR in Table 1. It should be noted that some studies had also focused on cross-position heterogeneity (sensors placed on different positions). The information related to cross-position heterogeneity are excluded from Table 1, as they are irrelevant to our work. In Table 1, “source-only model” represents a model which is directly trained based on source data.

From Table 1, it is clear that the existing literature mostly focuses on ADL and sport activities and there is a lack of a comprehensive study of potential heterogeneities in an occupational environment. In particular, most studies have considered different subjects, where the improvements are often modest (2.0% and 4.7%) and sometimes notable due to grouping subjects into more heterogeneous groups.

## 3. Methods

### 3.1. Experimental Design and Data Collection

The experimental sessions were designed to simulate common activities of ELWs. ELWs often work at work sites where lone work may occur and direct supervision is not feasible. Serious injury and fatality (SIF) hazard potentials that these lone workers may encounter include sudden illness, such as heart attack, occupational injuries, such as fall from an elevation, electrocution and slips or falls, and contributing factors to loss, such as high voltage and confined spaces [59]. Thus, the HAR approach developed by this study has an enormous potential to reduce injuries in ELWs. Furthermore, a subset of the tasks performed by ELWs are commonly explored in the context of both ADL and occupational HAR studies. This should make our results potentially generalizable to studies/applications where only a subset of our tasks are required.

To simulate ELWs’ tasks, we considered two groups of subjects, Group 1 and Group 2, each including 10 subjects (their anthropometric information is summarized in Table 2). Group 1 simulated the activities of ELWs through a scenario called separate task scenario and Group 2 performed the same activities using two scenarios of separate task scenario and mixed task scenario (the two groups had two subjects in common). Group 1 was used to study cross-sensor, cross-subject, and cross-sensor and cross-subject heterogeneities. Group 2 was used to evaluate cross-scenario heterogeneity, since Group 2 performed both separate and mixed task scenarios. The experimenters completed training and received project approval from University at Buffalo Institutional Review Board (IRB) and written informed consent was provided by all of the subjects.

The subjects were equipped with an Empatica E4 wristband (Empatica, Boston, United States, we call it Sensor 1) and a Maxim wristband (Maxim Integrated, San Jose, United States, we call it Sensor 2) on the wrist of their dominant hand to collect triaxial acceleration. We prefer acceleration-based measurements to biometric-based measurements, such as heart rate, as changes in biometric measurements are often delayed compared to the task start time [60]. Thus, biometric-based information is not considered suitable for detecting short duration tasks, which are commonly performed by electrical line workers. The sampling rate of Sensor 1 was 32 Hz and Sensor 2 collected the acceleration components at unevenly spaced timestamps which is common for wearable sensors [36]. Subjects then performed either the separate and mixed task scenarios as shown in Figure 2. For the mixed task scenario, the subjects kept repeating the circuit for 1 h, with in total around 7 replications. The Sensor 1 and Sensor 2 used Bluetooth to connect to a smartphone in the pocket of the subject and a tablet in the proximity of the subject, respectively. The data collection for Sensor 1 was operated by the Empatica *E4 realtime* Android app, where the data were stored in the device and transmitted to the cloud at the end of the experiment, while the same task for Sensor 2 was operated by a separate Android app. The start and end of each activity was annotated by an observer during the experiment.

### 3.2. Data Pre-Processing

Both Sensor 1 and Sensor 2 measure continuous gravitational force (g) exerted to each of the three axes. The measurement unit of Sensor 1 is g/64 (64 is equivalent to 1g), while the measurement unit of Sensor 2 is 1g. Both units were converted to 1 m/s^2^ to be consistent. As mentioned in Section 3.1, the sampling rate of Sensor 1 was 32 Hz, while Sensor 2 did not have a constant sampling rate over time. A linear interpolation was applied to the measurements of Sensor 2 to obtain a sampling rate of 32 Hz and deal with the sampling rate inconsistency. Linear interpolation was also helpful to deal with the missing data issue that occurred for Sensor 2. However, the interpolation was not carried out when the length of missing part was more than 1 s to avoid generating valueless synthetic data.

The non-overlapping fixed-size windowing technique was then employed to segment the labelled acceleration data, due to its efficiency and simplicity [61]. A window length of 10 s, equivalent to 320 acceleration datapoints, was considered for the segmentation of acceleration data collected from Group 1, as it was practical in our case and has been shown to result in accurate predictions [62]. The acceleration data from Group 2 were segmented using a window length of 4 s, equivalent to 128 acceleration datapoints, since the mixed task scenario performed by Group 2 involved single repetitions of the repetition-based activities, and in many cases a single repetition was shorter than 10 s (the same windowing was used for the separate task scenario data collected from Group 2 for consistency).

### 3.3. Feature Extraction

**Figure 3:**
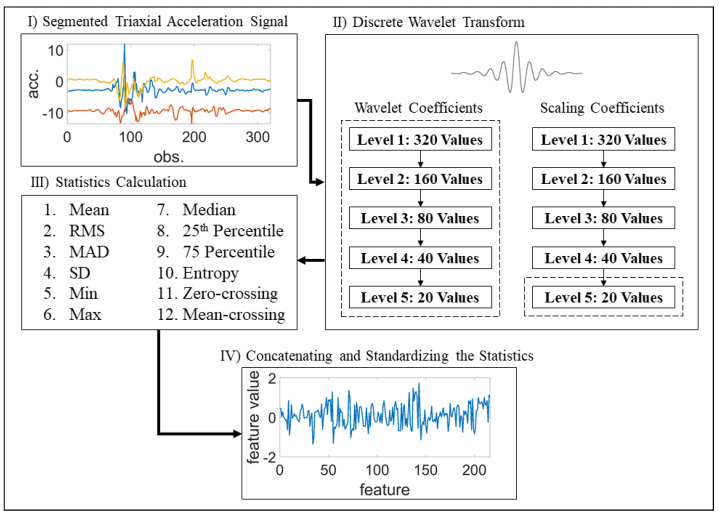
An overview of the feature extraction procedure.

Discrete wavelet transform (DWT) was employed to obtain the time-frequency domain features, as DWT has been shown to produce better results compared to time domain and frequency domain features when applied to a similar dataset [62]. In particular, following Lamooki et al. [62], a Daubechies-4 (d4) wavelet with 5 levels was used to decompose the segmented triaxial signals into scaling and wavelet coefficients. To reduce the number of input variables, 12 statistics, including mean, root mean square (RMS), mean absolute deviation (MAD), standard deviation (SD), minimum, maximum, median, 25th percentile, 75th percentile, entropy and number of zero- and mean- crossings were obtained for the wavelet coefficients in all 5 levels and for the scaling coefficients in the last level. The statistics calculated for the triaxial signals were then concatenated to create the time-frequency features with a length of 216. Finally, a standardization was applied to the time-frequency features, where the features were centered and scaled to have mean = 0 and standard deviation = 1. An overview of the feature extraction procedure applied to a 10-second window is presented in Figure 3.

### 3.4. Geodesic Flow Kernel for Domain Adaptation

Here, we used GFK to project the original features into a low-dimensional subspace. Unlike principal component analysis (PCA), GFK can project source and target features to common subspaces that inherit characteristics of both domains. A very important advantage of the GFK method is that it is equipped with a built-in automatic source selection procedure based on a metric, called rank of domain (ROD), which can be very helpful for selecting the subjects that are more likely to adapt well to the target subject. Furthermore, unlike other conventional DA approaches, such as transfer component analysis (TCA) and subspace alignment (SA), the only parameter of the GFK method can be tuned automatically. Here, we present a brief introduction and refer the interested reader to Gong et al. [38] for more details.

Assume that ϕ(0) and ϕ(1) are two points on Grassmann manifold corresponding to source and target data. Let $P_{S},P_{T}\in R^{D\times d}$ be two sets of basis of the subspaces belonging to the source and target domain, respectively, and $R_{S}\in R^{D\times (D-d)}$ be the orthogonal complement of $P_{S}$, where *d* denotes the dimensionality of the subspace. Using the canonical Euclidean metric for the Riemannian manifold, a geodesic flow can be constructed between ϕ(0) and ϕ(1) as

(1) $$\phi \left(t\right)=P_{S}U_{1}\Gamma \left(t\right)-R_{S}U_{2}\Sigma \left(t\right),$$

where *t* parameterizes a smooth curve between ϕ(0) and ϕ(1) and $U_{1}$ and $U_{2}$ are two orthonormal matrices obtained by the following singular value decompositions,

(2) $$P_{S}^{⊤}P_{T}=U_{1}\Gamma V^{⊤},R_{S}^{⊤}P_{T}=-U_{2}\Sigma V^{⊤}.$$

Γ and Σ are d×d diagonal matrices with diagonal elements $\cos\theta_{i}$ and $\sin\theta_{i}$ for i=1,…,d. In particular, $\theta_{i}$ represent principal angles between $P_{S}$ and $P_{T}$:

(3) $$0\leq \theta_{1}\leq \theta_{2}\leq \ldots \leq \theta_{d}\leq \pi /2$$

Principal angles quantify the overlapping degree of subspaces (we refer the interested reader to Drmac [63] for more details on principal angles). Furthermore, Γ(t) and Σ(t) are diagonal matrices with diagonal elements $\cos(t\theta_{i})$ and $\sin(t\theta_{i})$, respectively,

The geometric flow ϕ(t) embeds the data in Riemannian manifold and represents the incremental changes between source and target data. Let x be a feature vector from the source domain. $\phi {\left(t\right)}^{⊤}x$ projects x to the subspace ϕ(t). If *t* is close to 1, then the projected feature vector will resemble more the target domain, and vice versa for *t* close to 0. Hence, this projection would result in a set of features that inherit characteristics of both source and target domains. Consequently, a classifier which learned from the projected source features would perform well on the target features.

The selection of *t* or which set of *t* still remains a concern and Gong et al. [38] suggests to include all of them. Projecting the feature vectors into all subspaces $[\phi {\left(0\right)}^{⊤},\ldots ,$ $\phi {\left(t\right)}^{⊤},\ldots ,\phi {\left(1\right)}^{⊤}{]}^{⊤}x$ is explicitly not achievable, and Gong et al. [38] proposed an approach robust to any variation that inclines to either the source or the target or in between. Computationally, two feature vectors $x_{i}$ and $x_{j}$ are projected into ϕ(t) for a continuous *t* from 0 to 1 and then concatenated into infinite-dimensional feature vectors $z_{i}^{\infty }$ and $z_{j}^{\infty }$. The inner product between $z_{i}^{\infty }$ and $z_{j}^{\infty }$ gives the geodesic-flow kernel,

(4) $$\langle z_{i}^{\infty },z_{j}^{\infty }\rangle =\int_{0}^{1}{\left(\phi {\left(t\right)}^{⊤}x_{i}\right)}^{⊤}\left(\phi {\left(t\right)}^{⊤}x_{j}\right)dt=x_{i}Gx_{j},$$

where $G\in R^{D\times D}$ is a positive semidefinite (PSD) matrix. This operation can reduce the computational burden thanks to the kernel trick [38]. The conventional ML algorithms then apply this kernel to obtain domain-invariant low-dimensional representations. In our case, we use support vector machine (SVM) as a classifier.

An advantage of the GFK method is that it does not require any parameter tuning and its only free parameter *d* can be automatically selected using a subspace disagreement measure (SDM). To calculate SDM, the PCA subspaces of source data, target data and combined source and target data, ${\mathrm{PCA}}_{S}$, ${\mathrm{PCA}}_{T}$ and ${\mathrm{PCA}}_{S+T}$ are calculated. By intuition, if the two datasets resemble one another, then all three subspaces should be similar on the Grassmannian. The SDM exploits this notion and is formulated using the principal angles:

(5) $$D\left(d\right)=0.5\left[\sin\alpha_{d}+\sin\beta_{d}\right],$$

where $\alpha_{d}$ and $\beta_{d}$ represent the *d*-th principal angle between ${\mathrm{PCA}}_{S}$ and ${\mathrm{PCA}}_{S+T}$ and between ${\mathrm{PCA}}_{T}$ and ${\mathrm{PCA}}_{S+T}$, respectively. A small value of D(d) denotes small values of $\alpha_{d}$ and $\beta_{d}$, which indicates that ${\mathrm{PCA}}_{S}$ and ${\mathrm{PCA}}_{T}$ are well-aligned at the *d*-th dimension. Gong et al. [38] adopted a greedy algorithm to perform this optimization and select *d*.

**Figure 4:**
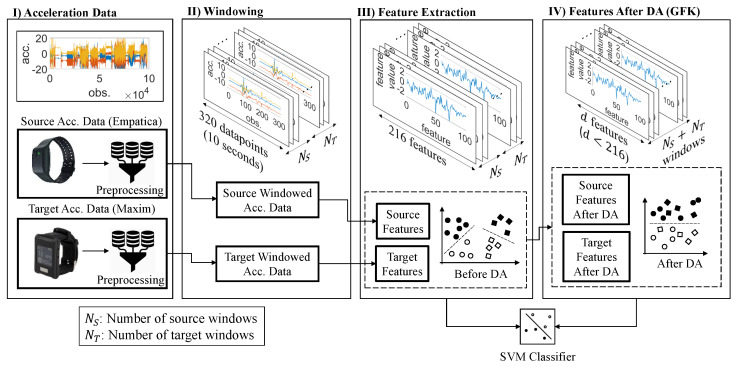
An illustration of the methodology for the cross-sensor case.

Finally, Gong et al. [38] developed an ROD metric, which is used to determine which datasets would result in the best adaptation to the target data without performing the domain adaptation and training the classifiers. In particular, ROD is computed for a pair of domains R(S,T) and two domains with a lower ROD are more likely to adapt well. Due to space limitation, we refer the interested reader to Gong et al. [38] for deeper details and rigorous mathematical analysis on ROD. Figure 4 gives an illustration of the methodology, where the cross-sensor case is used as an example.

## 4. Results

### 4.1. Cross-Sensor

For each subject from Group 1, the data collected from Sensor 1 and Sensor 2 were considered to be source data and target data, respectively. GFK was applied to the source and target data, which resulted in the representations of two datasets in a lower dimension. SVM was then employed to learn a classifier based on the transformed source data and applied to predict the labels associated with the transformed target data. On the other hand, our benchmark method assumes that the source data are not distributionally different from the target data. Thus, in this case, the SVM method was directly applied to train the classifier and predict the labels related to the target data.

We present the results for task-specific F1 scores, overall F1 score and overall accuracy for different subjects in Table 3 (SX-DA represents the results for the X-th subject when a DA is applied prior to using the SVM, while SX shows the results when no DA is employed). For the benchmark method (without DA), sometimes no value has been reported for the F1 scores associated with some of the tasks. In those cases, those specific tasks were fully misclassified. From overall F1 score and overall accuracy results, it is clear that DA improves the classification performance. In particular, the accuracy improvements for different subjects ranged from 0.08 to 0.42 with an average of 0.29.

### 4.2. Cross-Subject

Here, we only used the data collected from Sensor 1 worn by Group 1 subjects. For each subject, we considered them to be the target data and the remaining subjects to be the potential source data. The ROD metric was then employed to determine the *k* subjects from the potential source data that are most adaptable to the target data. In particular, the ROD metric was calculated for the target data paired with any remaining subject. The potential source subjects associated with the *k* lowest RODs were considered to be the source data. For the benchmark method, those *k* subjects were selected randomly. Then, similar to the cross-sensor case, a DA integrated with an SVM was used to predict the target labels and compared with the benchmark method, which only used the SVM.

**Figure 5:**
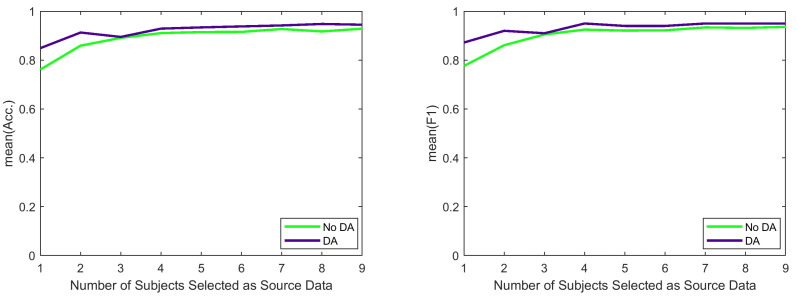
Impact of *k* on overall accuracy and overall F1 score in cross-subject classification.

The results for k=5 are presented in Table 4 and the results of a sensitivity analysis for the effect of *k* are illustrated in Figure 5. In Table 4, the $R\bar{O}D$ column shows the average of all of the ROD values associated with each subject, where a lower value of $R\bar{O}D$ for a specific target subject indicates that its selected source subjects are more adaptable to that target subject. Based on Table 4, using DA generally improves the overall accuracy and overall F1 score. However, the improvements in the cross-subject classification are not as large as those of cross-sensor classification. In particular, the accuracy improvements for different subjects ranged from −0.03 (a negative value indicates decrease in the accuracy) to 0.13 with an average of 0.02. Moreover, both DA and without DA methods did not perform well when applied to Subject 7. We could have guessed this poor performance even without performing the DA and learning the classifier because the highest $R\bar{O}D$ is related to Subject 7, which indicates that the selected source subjects associated with Subject 7 can not be well-adapted to that subject.

Finally, Figure 5 shows how *k* impacts the average of the accuracy and F1-score over all of the tasks and all of the subjects. To obtain the average of the F1-score, we had to ignore the cases where no values had been reported for the F1 scores associated with some of the tasks (due to misclassifying all of the observations associated with those tasks). Table 5 shows the number of those excluded cases for each *k* and method. Based on Figure 5, for different values of *k*, DA always achieves higher accuracies and higher F1 scores compared to the method without DA and a moderate value of *k* is sufficient for DA to achieve its highest F1 score.

### 4.3. Cross-Sensor and Cross-Subject

For each subject from Group 1, the data collected from Sensor 2 for that specific subject was considered to be the target data and the data collected from Sensor 1 for the remaining subjects was used as the potential source data. The selection of source subjects was performed similar to the cross-subject case (based on ROD metric and random selection for DA method and without DA method, respectively) and a DA integrated with an SVM was benchmarked against an SVM.

The comparative results for k=5 are given in Table 6. The accuracy improvements for different subjects ranged from 0.07 to 0.39 with an average of 0.24. Similar to the cross-subject case, the DA method (also the benchmark) does not show a good performance when applied to Subject 7 due to the lower adaptability of the selected source subjects to Subject 7. Moreover, $R\bar{O}D$ values for different subjects ranged from 0.030 to 0.051 with an average of 0.036, which were higher than those of cross-sensor case (ranging from 0.015 to 0.047 with an average of 0.021). This result makes sense because adaptation for a cross-sensor and cross-subject (simultaneously) case should be harder than that of just the cross-subject case.

**Figure 6:**
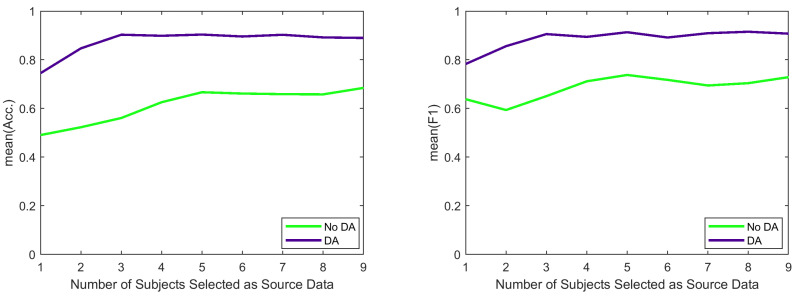
Impact of *k* on overall accuracy and overall F1 score in cross-sensor and cross-subject classification.

Finally, Figure 6 and Table 7 represent the sensitivity analysis based on different values of *k*. It is evident that DA always improves the overall accuracy and the overall F1 score and a low value of *k* (around 3) is sufficient for the DA method to achieve its highest accuracy and F1 score.

### 4.4. Cross-Scenario

In this case, the data collected from Sensor 1 worn by Group 1 were considered to be source data (separate task scenario) and the data collected from Sensor 1 worn by Group 2 were considered to be target data (mixed task scenario). As described in Section 3.1, the design of the separate task scenario followed the conventional activity recognition experiments, where the tasks were performed separately. On the other hand, mixed task scenario aimed to mimic a more realistic scenario, where the tasks were spread over the experiment.

Table 8 shows the results related to an SVM method equipped with DA benchmarked against an SVM without DA (the typing task was excluded here because it was not performed in the mixed task scenario). It is clear that DA does not improve the overall accuracy or overall F1 score.

## 5. Discussion

### 5.1. Summary of the Main Contributions

In this paper, we examined the potential heterogeneities in the occupational environment of ELWs and their impact on HAR algorithms, which is a prerequisite for promoting transferability of activity recognition models. We designed an experimental lab study to assess four research questions that pertain to transferability of activity recognition models in (1) cross-sensor, (2) cross-subject, (3) joint cross-sensor and cross-subject, and (4) cross-scenario heterogeneities.

**Cross-sensor.** We have shown that the information learned from a specific wearable sensor can not be directly used to perform activity recognition based on a new wearable sensor. First, there were inconsistencies between different sensors in measurement units and sampling rates. Further, there was missing data in the acceleration data collected from Sensor 2. In the preprocessing step, we resolved these preliminary inconsistencies using basic statistical techniques, such as interpolation. Second, the features extracted from the source data were distributionally different from those of the target data. The comparative analysis shown in Table 3 confirms this distributional heterogeneity, as a domain adaptation prior to an SVM could increase the classification accuracy by at least 0.08 (and on average 0.29) compared to when an SVM without DA is employed. This result is in accordance with Zhou et al. [36], where their deep domain adaptation framework could increase the classification accuracy by at least 0.04 (and on average 0.28) compared to an SVM baseline in cross-sensor classification of gesture and sport activities. Therefore, it is of utmost importance to assess the cross-sensor heterogeneity when a change or update occurs in the configuration of the activity recognition system and, if needed, employ a DA method to maintain a good performance.

**Cross-subject.** The comparative analysis summarized in Table 4 demonstrated that the models directly trained (without DA) using a limited number of subjects could be used to recognize the activities of a new unseen subject with an acceptable level of accuracy. However, on average, the classification accuracy was improved by 0.02 through applying DA and transferring the information from a subset of the pre-existing subjects, selected by ROD, to a new subject. The accuracy improvement in this case was not as large as that for the cross-sensor case. This result conforms with the accuracy improvement results reported in the literature for cross-subject heterogeneity. For example, Zhao et al. [55] and Chakma et al. [37] achieved accuracy improvements of 0.05 and 0.02, respectively. Hosseini et al. [54] and Zhou et al. [36], however, had reported higher accuracy improvements for cross-subject heterogeneity, as there were higher heterogeneities between their subjects in source data and target data. In particular, Hosseini et al. [54] had grouped the subjects into adults and children and Zhou et al. [36] had grouped the subjects by age, body mass index (BMI), and sex.

In this case, the correlation between overall accuracy and $R\bar{O}D$ was −0.83. Gong et al. [38] used ROD for image classification and demonstrated that ROD correlates well with recognition accuracies on the target domains and can reliably identify the best source domains to adapt (a lower ROD indicates stronger adaptability of the source domain to the target domain). The significant correlation value obtained in this case confirms the usability of ROD metric in the source selection process of activity recognition. Therefore, one can use ROD to select a limited number of source subjects among many subjects and avoid less adaptable subjects. In addition to improving performance, it is computationally more efficient to select a few subjects that are likely to adapt well to the target subject, rather than trying each one [38]. This advantage can promote developing fast-response IoT wearable sensors, which are essential for people working in hazardous environments [64].

As mentioned in Section 4.2, DA did not perform well when applied to Subject 7 and we could have foreseen this bad performance without performing the DA and prediction, as the $R\bar{O}D$ value related to this subject is higher compared to other subjects. Although our approach attempts to select the most adaptable subjects as source data, it was hard to find subjects that could be well-adapted to Subject 7. Based on our recorded demographic information, Subject 7 was a female who was 163 centimeters tall and weighed 44 kilograms. Her BMI was 16.56 kg/m^2^, which was lower than other subjects. Therefore, the resulting poor performance could be explained. Given a higher number of subjects, we expect that a clustering approach based on ROD metric could have improved the accuracy for this specific subject and the overall accuracy of our approach.

**Joint cross-sensor and Cross-subject.** The results of the joint cross-sensor and cross-subject case were along the same lines of when we dealt separately with the cross-sensor or cross-subject cases, apart from the fact that simultaneous existence of two heterogeneities made the problem harder. The accuracy improvement resulting from DA ranged from 0.07 to 0.39, with an average of 0.24. The average of $R\bar{O}D$ values was 0.036, which was higher than the 0.021 observed in the cross-sensor case. This result indicates that when two heterogeneities co-existed, the adaptation was harder. After DA, the overall accuracy averaged over all of the subjects was 0.90, which was lower than the average overall accuracy of cross-sensor (0.95) and cross-subject (0.93) cases. Similar to the cross-subject case, a meaningful correlation between $R\bar{O}D$ and obtained overall accuracies (−0.74) and a high $R\bar{O}D$ value for Subject 7 confirms the usability of ROD metric as a source selection approach. In summary, leveraging DA methods is of great importance when dealing with a joint cross-sensor and cross-subject situation.

**Cross-scenario.** We demonstrated that the information learned from a controlled lab experiment (separate task scenario) can be directly applied to another scenario, which is more similar to the environment of a real-world workplace from task dispersion viewpoint (mixed task scenario). In particular, Table 8 shows that both accuracy and F1 score are acceptable before DA and decrease after DA. This result indicates that the new representation of the features in a lower dimension is not more informative than the original features and results in destruction of important information. We conclude that there is not a significant heterogeneity between separate and mixed task scenarios. This conclusion is in agreement with Hong et al. [31], who showed that the accuracy of their ADL recognition for field (in-home) and lab studies were comparable.

### 5.2. Limitations and Suggestions for Future Research

There are a few limitations that must be noted for this work. First, our lab experiment was not designed to overexert the subjects and induce fatigue in them. In laborious jobs, there is a possibility that the distribution of the extracted features changes over time due to fatigue, which may impact the performance of activity recognition. To investigate this, one can relax the distributional stationarity assumption and assume that target data not only differ from source data, but differ from it in a continually progressing manner. For instance, Hoffman et al. [65] developed a continous manifold based adaptation approach for scene detection with gradually changing lighting. A similar approach can be employed to examine the impact of fatigue on classification, as fatigue usually evolves in an incremental way. Second, in our mixed task scenario, the activities were spread over the time of the experiment to imitate the situation of a real-world workplace. However, there might be other heterogeneity sources apart from dispersion of activities that can impact the performance of activity recognition, such as environmental factors. Thus, a more comprehensive study is required to identify other factors and examine how they can impact the performance of activity recognition.

Finally, we present three suggestions for future study. First, studies should investigate how HAR can benefit from an ROD-based clustering approach. The conventional belief is that demographically similar subjects would show similar activity patterns; however, Hong et al. [31] demonstrated that their approach, called single-personalization (SP), performed better than others that rely on subjects’ demographic information for classifying their activities. In particular, they showed that the subjects who matched each other based on SP were often demographically different. An ROD-based clustering method can group subjects into clusters, where the subjects within each cluster are more likely to have similar activity patterns. Second, real-time activity recognition is more favorable than offline activity recognition from a safety monitoring perspective. However, real-time DA is a challenging task, as DA requires adequate information about distribution of target domain, which is not available at the beginning of a real-time monitoring. For instance, some classes are absent at the beginning of the monitoring, which poses a serious challenge to DA. Real-time DA has been studied in multiple works [34,65,66] and is worthy of consideration in activity recognition. Third, unlabeled activities are important to consider. One way to deal with this issue is to treat unlabeled activities as a null class. However, this is challenging from a data collection viewpoint, as the null class should contain a wide range of activities, which are dissimilar to other existing activities. Thus, real-time classification of occupational activities when null activities exist can be an interesting direction of future work.

## 6. Conclusions

While wearable sensors offer favorable opportunities for activity recognition and monitoring of occupational workers, the performance of activity recognition remains a concern due to a number of real-world heterogeneities. In this work, we aimed to investigate the impact of four heterogeneity sources (cross-sensor, cross-subject, joint cross-sensor and cross-subject, and cross-scenario heterogeneities) on activity recognition performance of a common set of activities in electrical line workers. To that end, a support vector machine classifier equipped with a domain adaptation method was benchmarked against a standard support vector machine baseline. In addition, a metric, rank of domain, was used for the first time to automatically determine which existing subjects as training set would give us the best performance on a new unseen subject. Our results demonstrated that cross-sensor, cross-subject, and joint cross-sensor and cross-subject heterogeneities had an adverse impact on activity recognition performance, where domain adaptation alleviated the adverse impact and improved the accuracy. Cross-scenario heterogeneity, on the other hand, did not show any harmful impact on classification accuracy. We also uncovered the effectiveness of the rank of domain metric and verified its interpretability. We believe that our work can pave the way for applying activity recognition to real-world occupational environments, where heterogeneities exist.

## Figures and Tables

**Table 1:** A summary of human activity recognition studies using domain adaptation.

Reference	Area	Heterogeneity Source	Source and Target Data	Accuracy Improvement
[15]	ADL	Different Sensors	**Source:** Smart watch (phone) **Target:** Smart phone (watch)^1^	46.9% over the source-only model
[57]	ADL and sport activities	Different subjects	**Source:** Subject X **Target:** Subject Y	23.3% over the source-only model per their first experiment
[54]	ADL	Different ages	**Source:** Adult **Target:** Children	16.2% improvement in F1-score over the source-only model
[36]	Gesture and sport activities	1. Different sensors 2. Different subjects	1. **Source:** Sensor X **Target:** Sensor Y 2. **Source:** Group X (based on age, sex. and BMI) **Target:** Group Y (based on age, sex, and BMI)	1. 27.7% over source-only model 2. 19.9% over source-only model
[55]	ADL and sport activities	Different subjects	**Source:** Subject(s) X **Target:** Subject(s) Y ^2^	4.7% over source-only model
[37]	ADL	Different subjects	**Source:** Subjects X and Y **Target:** Subject Z ^3^	2.0% over source-only model

^1^ Either **Source:** Smart watch and **Target:** Smart phone or **Source:** Smart phone and **Target:** Smart watch; ^2^ Subject(s) Y represents Subject Y (one subject) or Subjects Y (a group of subjects); ^3^ Subjects X and Y represent two subjects and Subject Z represents one subject.

**Table 2:** Summary of anthropometric information for the subjects. The values in columns 3–5 are the average. (standard deviation).

Group	Gender	Count	Age	Body Mass (kg)	Height (m)
1	Male	6	26.83 (4.30)	87.45 (18.70)	1.81 (0.10)
	Female	4	21.25 (4.55)	57.88 (19.12)	1.59 (0.05)
2	Male	6	26.67 (3.30)	81.53 (18.03)	1.80 (0.07)
	Female	4	21.00 (1.22)	59.12 (14.71)	1.62 (0.08)

**Table 3:** Cross-sensor classification performance. Abbreviations: EP: electrical panel, H: hoisting, Ld: ladder, Lf: lifting; OH: overhead; P: pushing; St: sitting; Sd: standing; Tp: typing; W: walking (For some of the cases, “-” has been reported as task-specific F1 score, which means that the denominator of the precision or recall for that specific case was zero.)

Subject	Task-Specific F1 Score in the Test Data										$\bar{F1}$	$A\bar{c}c.$
	EP	H	Ld	Lf	OH	P	St	Sd	Tp	W		
S1	0.81	1.00	0.22	0.72	0.58	0.98	-	1.00	0.65	1.00	-	0.76
S1-DA	1.00	0.96	0.93	0.92	1.00	1.00	1.00	1.00	1.00	1.00	0.98	0.97
S2	0.90	1.00	0.94	0.70	0.87	0.96	0.90	0.91	1.00	-	-	0.87
S2-DA	1.00	1.00	0.94	0.84	0.97	1.00	1.00	1.00	0.97	0.86	0.96	0.95
S3	0.85	0.41	0.68	0.50	0.87	-	-	1.00	0.46	0.52	-	0.56
S3-DA	1.00	0.98	0.98	0.98	1.00	0.58	0.97	1.00	0.71	0.97	0.92	0.92
S4	0.94	0.90	0.21	0.47	1.00	0.88	-	0.86	0.60	-	-	0.62
S4-DA	1.00	1.00	0.80	0.78	1.00	1.00	0.74	1.00	0.79	1.00	0.91	0.90
S5	1.00	0.73	0.15	0.47	1.00	0.98	-	1.00	0.67	-	-	0.63
S5-DA	1.00	1.00	0.99	1.00	1.00	0.98	0.90	1.00	0.92	1.00	0.98	0.98
S6	0.80	0.77	0.35	0.70	1.00	0.59	-	0.97	0.67	-	-	0.64
S6-DA	1.00	0.96	0.94	0.96	1.00	1.00	0.58	0.97	0.77	1.00	0.92	0.93
S7	0.45	0.09	0.75	0.58	1.00	-	-	0.97	0.43	-	-	0.54
S7-DA	1.00	0.95	0.95	0.89	1.00	1.00	0.94	1.00	0.94	0.90	0.96	0.96
S8	0.52	0.91	0.85	0.09	1.00	-	0.21	0.73	0.65	0.11	-	0.63
S8-DA	1.00	0.91	0.96	0.88	1.00	0.91	1.00	1.00	0.91	1.00	0.96	0.96
S9	0.76	0.89	0.60	0.57	1.00	0.91	-	0.86	0.62	-	-	0.65
S9-DA	0.94	0.95	0.96	0.90	1.00	0.98	1.00	0.90	1.00	0.97	0.96	0.96
S10	1.00	0.13	0.85	0.62	1.00	0.67	-	1.00	0.64	0.58	-	0.70
S10-DA	1.00	0.92	0.89	0.76	1.00	0.90	1.00	1.00	0.97	1.00	0.94	0.93

**Table 4:** Cross-subject classification performance. Abbreviations: EP: electrical panel, H: hoisting, Ld: ladder, Lf: lifting; OH: overhead; P: pushing; St: sitting; Sd: standing; Tp: typing; W: walking (For some of the cases, “-” has been reported as task-specific F1 score, which means that the denominator of the precision or recall for that specific case was zero.)

Subject	Task-Specific F1 Score in the Test Data										$R\bar{O}D$	$\bar{F1}$	$A\bar{c}c.$
	EP	H	Ld	Lf	OH	P	St	Sd	Tp	W			
S1	1.00	1.00	1.00	0.98	1.00	1.00	0.90	1.00	0.92	0.97	-	0.98	0.98
S1-DA	1.00	1.00	0.95	0.92	1.00	1.00	0.94	1.00	0.94	1.00	0.019	0.98	0.97
S2	0.94	0.98	0.63	0.66	0.94	1.00	0.90	0.97	0.89	1.00	-	0.89	0.86
S2-DA	0.97	1.00	0.66	0.72	1.00	0.94	1.00	1.00	0.97	1.00	0.015	0.93	0.89
S3	1.00	0.93	0.98	0.92	1.00	0.96	1.00	0.97	1.00	0.94	-	0.97	0.97
S3-DA	1.00	0.98	0.98	0.98	1.00	0.98	1.00	1.00	0.97	1.00	0.019	0.99	0.99
S4	1.00	0.95	0.96	0.94	1.00	1.00	1.00	1.00	1.00	1.00	-	0.99	0.98
S4-DA	1.00	1.00	0.94	0.91	1.00	1.00	0.94	1.00	0.94	1.00	0.018	0.97	0.97
S5	1.00	1.00	1.00	1.00	1.00	0.98	0.94	1.00	0.90	1.00	-	0.98	0.99
S5-DA	1.00	1.00	1.00	1.00	1.00	1.00	1.00	1.00	1.00	1.00	0.018	1.00	1.00
S6	1.00	1.00	0.99	0.98	1.00	1.00	0.92	1.00	0.90	1.00	-	0.98	0.98
S6-DA	1.00	0.98	0.99	1.00	1.00	1.00	0.97	1.00	0.97	1.00	0.022	0.99	0.99
S7	0.36	0.86	0.83	0.71	0.52	0.15	0.86	-	0.94	1.00	-	-	0.67
S7-DA	-	0.76	0.90	0.73	0.45	0.48	0.83	0.74	0.77	1.00	0.047	-	0.72
S8	1.00	0.14	0.92	0.58	1.00	0.90	0.79	1.00	0.84	1.00	-	0.82	0.83
S8-DA	1.00	0.94	0.94	0.86	1.00	0.97	1.00	1.00	1.00	1.00	0.020	0.97	0.96
S9	0.92	0.98	1.00	0.84	0.90	1.00	1.00	0.80	1.00	1.00	-	0.94	0.94
S9-DA	0.80	1.00	0.99	0.83	0.90	1.00	1.00	0.80	0.92	1.00	0.017	0.92	0.93
S10	0.97	1.00	0.95	0.89	1.00	0.90	1.00	0.97	0.97	0.87	-	0.95	0.95
S10-DA	0.94	1.00	0.81	0.82	1.00	0.88	1.00	1.00	0.94	1.00	0.017	0.94	0.92

**Table 5:** The number of excluded cases to obtain overall F1 score for cross-subject classification (totally, there were 10Subjects×10Tasks=100Cases for each method).

Method	k = 1	k = 2	k = 3	k = 4	k = 5	k = 6	k = 7	k = 8	k = 9
No DA	4	1	2	2	1	1	1	2	1
DA	2	0	1	2	1	0	1	1	1

**Table 6:** Joint cross-sensor and cross-subject classification performance. Abbreviations: EP: electrical panel, H: hoisting, Ld: ladder, Lf: lifting; OH: overhead; P: pushing; St: sitting; Sd: standing; Tp: typing; W: walking (For some of the cases, “-” has been reported as task-specific F1 score, which means that the denominator of the precision or recall for that specific case was zero).

Subject	Task-Specific F1 Score in the Test Data										$R\bar{O}D$	$\bar{F1}$	$A\bar{c}c.$
	EP	H	Ld	Lf	OH	P	St	Sd	Tp	W			
S1	0.67	0.63	0.63	0.56	0.94	0.44	-	1.00	0.67	-	-	-	0.61
S1-DA	1.00	1.00	1.00	1.00	1.00	1.00	0.52	1.00	0.76	1.00	0.039	0.93	0.94
S2	1.00	1.00	-	0.46	0.94	0.61	-	0.74	0.67	0.55	-	-	0.59
S2-DA	0.92	1.00	0.86	0.84	0.97	0.98	0.69	1.00	0.77	1.00	0.030	0.90	0.90
S3	0.13	0.93	0.99	0.84	0.72	0.22	-	1.00	0.48	-	-	-	0.67
S3-DA	0.83	1.00	0.97	0.93	0.89	0.83	0.97	1.00	0.81	1.00	0.031	0.92	0.93
S4	0.92	0.52	0.89	0.75	1.00	0.95	-	1.00	0.60	1.00	-	-	0.81
S4-DA	1.00	1.00	0.92	0.84	1.00	1.00	0.94	1.00	0.92	1.00	0.031	0.96	0.96
S5	0.61	0.97	0.90	0.85	1.00	0.30	-	1.00	0.58	0.97	-	-	0.77
S5-DA	0.89	1.00	0.95	0.93	0.97	0.88	0.97	1.00	0.91	1.00	0.031	0.95	0.95
S6	0.96	1.00	0.96	0.88	0.97	1.00	-	0.97	0.67	0.87	-	-	0.88
S6-DA	1.00	1.00	0.92	0.86	1.00	1.00	0.87	0.97	0.89	1.00	0.032	0.95	0.95
S7	0.12	0.32	0.76	0.56	0.53	-	-	-	0.63	0.52	-	-	0.43
S7-DA	-	0.80	0.89	0.70	-	0.30	0.97	0.97	0.79	0.90	0.051	-	0.70
S8	0.89	0.33	0.84	0.56	0.94	1.00	-	1.00	0.65	-	-	-	0.72
S8-DA	0.88	0.45	0.84	0.96	1.00	0.83	0.97	1.00	0.97	0.94	0.031	0.88	0.89
S9	0.76	0.67	0.95	0.59	1.00	0.67	-	0.84	0.67	-	-	-	0.69
S9-DA	0.88	0.97	0.98	0.89	1.00	0.98	0.94	0.89	0.89	1.00	0.041	0.94	0.94
S10	0.27	0.63	0.48	0.63	0.65	-	-	0.76	0.63	-	-	-	0.49
S10-DA	0.69	0.98	0.89	0.91	1.00	0.80	0.83	1.00	0.71	0.94	0.044	0.87	0.88

**Table 7:** The number of excluded cases to obtain overall F1 score for cross-sensor and cross-subject classification.

Method	k = 1	k = 2	k = 3	k = 4	k = 5	k = 6	k = 7	k = 8	k = 9
No DA	32	20	23	21	19	19	15	16	16
DA	5	1	1	0	2	0	2	4	3

**Table 8:** Cross-scenario classification performance. Abbreviations: EP: electrical panel, H: hoisting, Ld: ladder, Lf: lifting; OH: overhead; P: pushing; St: sitting; Sd: standing; W: walking.

Method	Task-Specific F1 Score in the Test Data									$\bar{F1}$	$A\bar{c}c.$
	EP	H	Ld	Lf	OH	P	St	Sd	W		
No DA	0.64	0.94	0.91	0.90	0.95	0.84	0.97	0.98	1.00	0.90	0.93
DA	0.53	0.93	0.82	0.90	0.94	0.84	0.93	0.93	0.99	0.87	0.90

## Data Availability

To encourage future research and/or adoption of our work, we have made our **MATLAB** code available at https://github.com/sahand-hajifar/Occupational-Task-Recognition-via-Domain-Adaptation (accessed on 3 October 2021).
